# An event-related potential study of P300 in preschool children with attention deficit hyperactivity disorder

**DOI:** 10.3389/fped.2024.1461921

**Published:** 2024-11-13

**Authors:** Mengjiao Tao, Jing Sun, Siqi Liu, Yike Zhu, Yongying Ren, Ziqi Liu, Xiaoyue Wang, Wenmin Yang, Guannan Li, Xin Wang, Wei Zheng, Jianzhao Zhang, Jian Yang

**Affiliations:** ^1^ Department of Pediatric Neurology, Children’s Hospital Affiliated to Capital Institute of Pediatrics, Beijing, China; ^2^ Department of Pediatrics, Beijing Friendship Hospital, Capital Medical University, Beijing, China; ^3^ Center of Children’s Healthcare, Children’s Hospital Affiliated to Capital Institute of Pediatrics, Beijing, China; ^4^ Medical Technology Department, Peking University Sixth Hospital, Beijing, China; ^5^ Guangzhou Rainjet Medical Equipment Co., Guangzhou, Guangdong, China

**Keywords:** attention deficit hyperactivity disorder, event related potential, P300, children, ERP, ADHD

## Abstract

**Objective:**

To study the characteristics of event related potential P300 in preschool children with attention deficit hyperactivity disorder (ADHD) aged 4–6 years (4≤ age <6), and to explore the differences in cognitive function compared with healthy children. To explore a new method for the study of cognitive function in preschool children with ADHD.

**Methods:**

A total of 73 preschool children aged 4–6 years were selected from the outpatient clinic of Neurology Department, the ADHD-specialized Clinic and Health Care Department of the Children's Hospital Affiliated to the Capital Institute of Pediatrics from March 2021 to May 2024. They were divided into the ADHD group (45 cases) and healthy children group (28 cases). Event related potential P300 was measured in all children and the amplitude and latency of the wave were compared between the two groups separately.

**Results:**

The latency of P300 at all the recording electrodes (Fz, Cz, Pz, Oz, C3, C4) in the ADHD group was significantly longer than controls (*p* < 0.05). The wave amplitudes of children with ADHD were significantly higher than controls at Pz and Oz points.

**Conclusion:**

The differences between two groups in P300 test show that preschool children with ADHD present longer latency at central line of the brain and bilateral central lobes compared with healthy children, and higher amplitude at the central parietal lobe and central occipital lobe. It may suggest that attention cognition has already impaired in preschool children with ADHD.

## 1 Introduction

Mental disorders are becoming one of the biggest global health problems in children and adolescents, which is also a major factor in their overall life expectancy (1). Attention deficit hyperactivity disorder (ADHD) is the most common neurodevelopmental disorder in children and adolescents that manifests with symptoms of inattention, hyperactivity, and/or impulsivity in early childhood (2, 3). In recent years, a significant increasing number of preschool children with ADHD symptoms has been coming to clinical attention (4). It is reported that the prevalence of ADHD among school-age children is between 9 and 15 precent (5). However, many mental disorders are present during preschool years but most of them are not diagnosed until school age, including ADHD, the prevalence of which is estimated to be about 3% in preschool children (6). According to previous studies, nearly 90% of preschoolers with ADHD are not received a timely diagnosis, suggesting that they are less likely to receive effective and proper interventions that can lead to adverse outcomes (6, 7). In addition, the majority (more than 70%) of preschoolers with clinical issues about ADHD will persist throughout the school years and even into early adulthood, continuing to meet the diagnostic criteria for ADHD, which means children who show hyperactivity in the preschool years tend to develop impulsive behaviors, aggressive behaviors and social adjustment problems during the school years (8, 9). Consequently, it is very important to timely identify and assess the disorders in the preschool years of children with ADHD to improve their symptoms and the quality of life. Due to the instability of behavioral manifestations during preschool period, there is an imminent demand to find an objective predictor to assist clinicians in evaluating the condition of children.

Cognitive function refers to the advanced cognitive abilities necessary for the goal-directed behavior, which develops primarily during the preschool period (aged 3–5 years) (10). Current studies generally support that the cognitive function deficit is an essential contributor to the symptoms with ADHD, and this association is well-documented not only in school-age children but also in preschoolers (11–13). Therefore, timely identification of cognitive function deficit in children during their preschool years may contribute to early assessment and diagnosis of ADHD. Electrophysiological techniques, as a non-invasive measurement of brain state and neural activation, have been widely used in the studies to explore the cognitive functions due to their high temporal resolution and functional relevance (14).

Event-related potentials (ERPs) is one of the most broadly utilized techniques in non-invasive neurological testing today. Through time-locked mechanism, ERPs can reveal the changes in neuronal signals in milliseconds, providing a temporal and load measure of changes in subject's brain activity while processing events occurring in real time (15). P300 is an endogenous component of ERPs with a peak of approximately 250–600 ms and reflects the cognitive function of participants (16). In ADHD, a wide range of deficits in cognitive domains have commonly been found, including attention, inhibitory control and working memory. P300 is widely used in the study of ADHD and cognitive deficits. A lot of researches focusing on adults and school-age children have showed that ADHD patients performed differently in P300 compared with the healthy, which well reflects their varies in cognitive functions (17–19). As a result, P300 has been consistently considered to be an indicator of cognitive processes and an objective criteria for cognitive function, as well as a promising endophenotype for ADHD (20–22). The diagnosis of ADHD in preschool children still remains controversial but P300 could provide uncontroversial evidence for the assessment of cognitive deficits. Therefore, it is particularly important to conduct studies on P300 in younger children, which may not only improve the current situation in ADHD assessment, but also have a chance to advance the time of clinical intervention, thereby improving the prognosis of the child.

The present study was conducted to investigate whether there are differences in cognitive function between preschool children with ADHD and healthy preschoolers aged 4–6 years, by analyzing and comparing the characteristics of their P300 waves, with the aim of exploring new approaches for the study of cognitive function in preschool children with ADHD.

## 2 Methods

### 2.1 Subjects

The study was approved by the Ethics Committee of the Children's Hospital Affiliated to the Capital Institute of Pediatrics. As all the participants were minors, written informed consents were collected from their guardians in accordance with the Declaration of Helsinki.

We recruited a total of 93 preschool children aged 4–6 years, who attended junior to senior kindergarten classes, from Neurology Department, the ADHD-specialized Clinic and Health Care Department of the Children's Hospital Affiliated to the Capital Institute of Pediatrics from March 2021 to May 2024. All the controls were healthy children, recruited from Health Care Department. The diagnosis of ADHD was made by more than two doctors, at least one of whom was the chief physician. Of the children whose caretakes complained of hyperactivity and/or attention deficit, 20 participants were excluded for not meeting the diagnostic criteria for ADHD according to the Diagnostic and Statistical Manual of Mental Disorders, Fifth Edition(DSM-5) (2). As for the children, who were diagnosed with ADHD, the situation of each child was explained with the parents honestly and efforts were made to find out individualize treatment options including both pharmacological and non-pharmacological treatments. ADHD children were categorized into three groups (2 predominantly attention deficit, ADHD-I; 20 predominantly hyperactive/impulsive disorder, ADHD-HI; and 23 combined type, ADHD-C) based on DSM-5. Eventually, 73 subjects participated in the study (28 controls and 45 ADHD), and all the participants exhibited DQ (Developmental quotient) ≥70 based on the Neuropsychological Development Assessment Scale for Children Aged 0–6 years(version 2016), which is widely used in China to assess the developmental level of children aged 0–6 years. All children with ADHD were diagnosed for the first time and were not taking psychostimulant medications prior to evaluation. None of the participants suffered from other types psychiatric disorders (such as autism spectrum disorder, anxiety disorder, obsessive-compulsive disorder) or neurological system diseases (such as epilepsy, tic disorder, cerebral palsy). Severe head injuries were also excluded. All subjects had normal or corrected-to-normal hearing and vision.

### 2.2 Procedure

The entire experimental procedure was conducted in a sound-shielded and dimly lit room, without interference and lasted for 20–30 min. The body temperature of the participants was below 37 degrees Celsius. Children were instructed to sit quietly and not to make any other movements while the electrodes were attached. Before the trial started, the physician would explain the processes of the task and launched into a practice to ensure the participants could adequately understand and perform the task. During the experiment, children were seated in front of a 17 inch screen with the seat was approximately 50 cm from the center of the screen. A repeated trial would be conducted for the children with a low level of cooperation in the first trial (23 children had to conduct repeated trials, occupied 24.73%), and the results with a higher superposition rate were included in the analysis.

### 2.3 Stimuli and task

**Figure 1:**
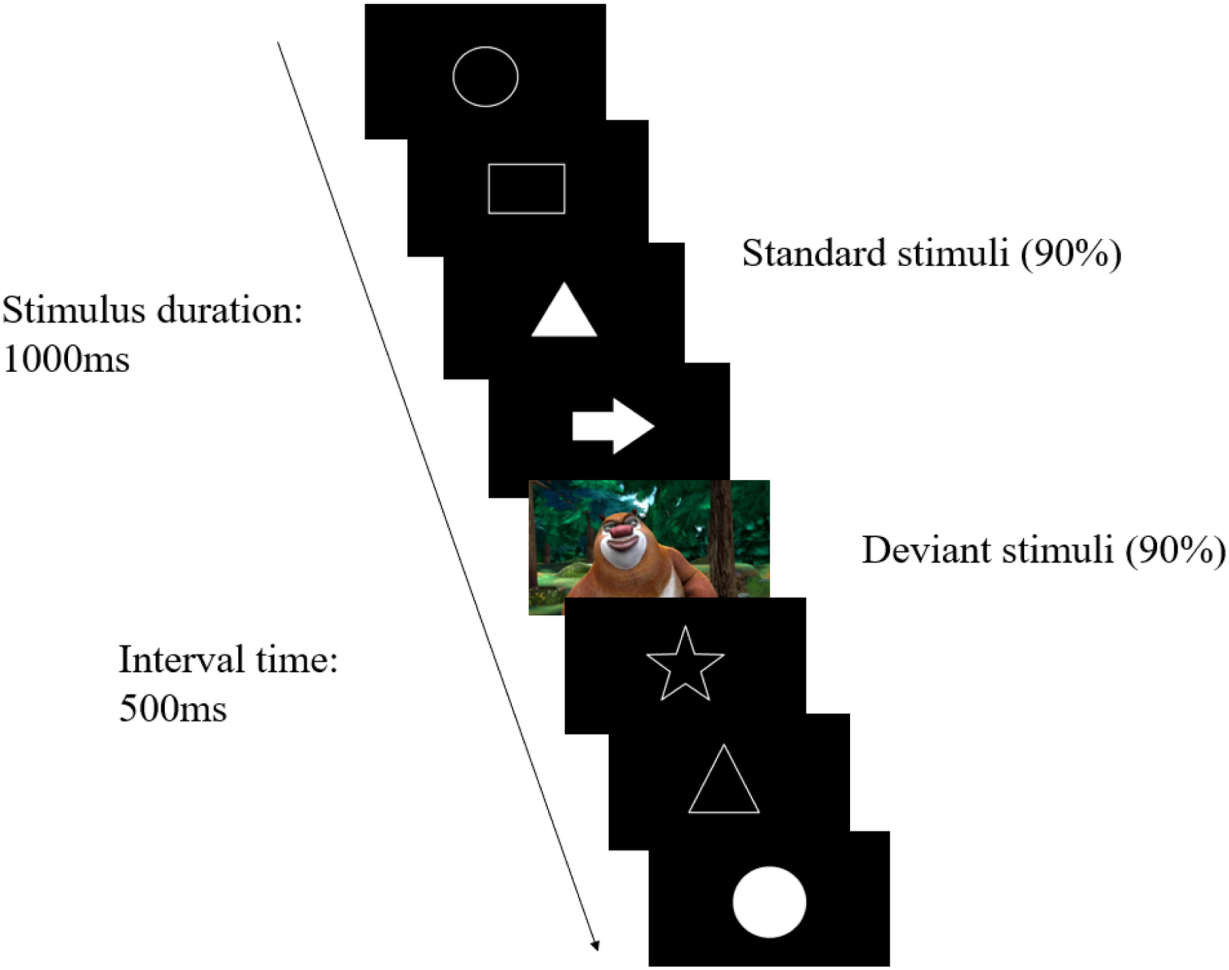
Visual oddball paradigm.

In our experiment, P300 was elicited by the visual Oddball paradigm (Figure 1), which was administered and displayed through the Innervate software. All the images appeared for 1,000 ms at the center of the screen on a black background, with a 500 ms interval. There were 40 images in total, of which 32 were different types of white patterns (standard stimuli) and 8 were colorful cartoon characters (deviant stimuli). The standard stimuli and deviant stimuli presented randomly with a ratio of 4:1. Participants were instructed to press the response button when the deviant-stimulus images occurred.

### 2.4 ERP recording and analysis

**Figure 2:**
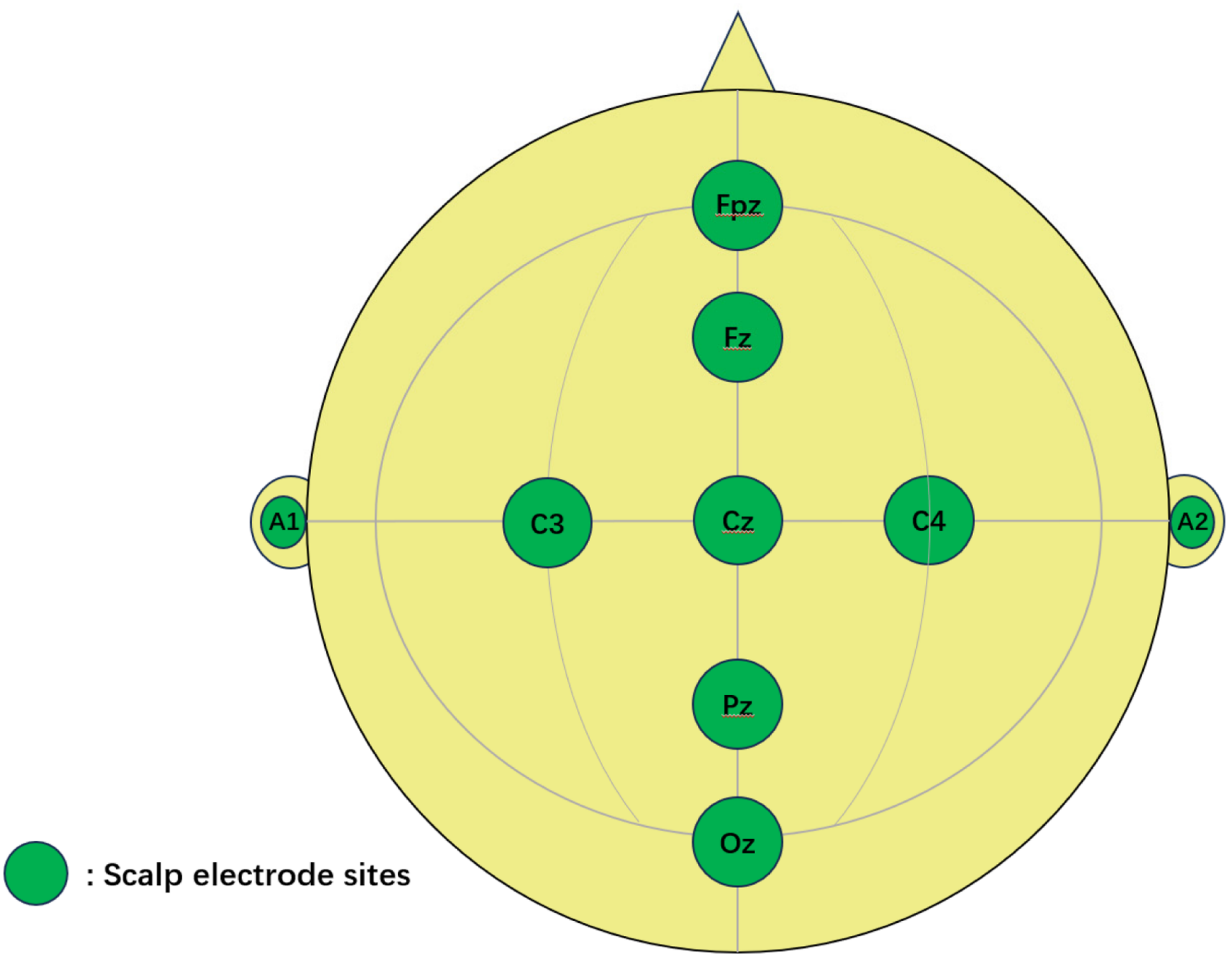
Schematic of the electrode sites.

All measurements were performed in the Neurological Function Laboratory of the Children's Hospital Affiliated to the Capital Institute of Pediatrics. The original data of P300 were measured and recorded by a medical event-related potentiometer (WJ-1A) manufactured by Guangzhou Rainjet Medical Co., LTD. The electroencephalographic activity was recorded at the centerline (Fz, Cz, Pz and Oz) and bilateral central parietal area electrode sites (C3 and C4) according to the 10–20 system using a soft electrode cap suitable for preschool children. The ground electrode was positioned at the frontal midline electrode (Fpz), and all the electrodes were referenced to the right ear electrode(A2) (Figure 2) with a sampling frequency of 1,024 Hz. The impedance of all recording electrode sites was kept below 30*Ω*. The pre-filter range was set to 0.1–70 Hz and the post-filter was 3–70 Hz. The latency and amplitude of P300 were recorded in this study: the latency was defined as the interval time from the start of the task to the appearance of the spike, and the amplitude referred to the voltage level of the wave crest, expressed as the height of the spike. If two identical crests occurred in one P300 wave, the intersection of the ascending branch and descending branch was defined as the crest point, otherwise, the higher crest point prevailed. In the event of the latency/amplitude was out of the range of normal values, regarded as an outlier and not included in the calculation.

### 2.5 Statistical analysis

Statistical analysis was conducted through IBM SPSS version 26.0. (Armonk, NY: IBM Corp). Normality test was performed for all continuous variables. Data confirming to normal distribution were expressed as mean ± standard deviation ($\bar{x}\pm s$), and the skewed continuous variables were expressed as median with interquartile range [M (P25, P75)]. As in the case of demographic data and ERP data, independent *t*-test or Wilcoxon rank-sum test were performed for comparison between groups of measurement data. ANOVA was used to compare the measurement data of the three groups following normal distribution and homogeneity of variance. And categorical variables, such as gender, were compared using the chi-square test. All statistical tests were two-sided with a significance level of 0.05.

## 3 Results

### 3.1 Baseline characteristics

A total of 73 subjects were included in this study, and the baseline characteristics are shown in Table 1. The median age in both groups was [4 (4, 5)] years. There was no significant difference in ages between the two groups (*p* > 0.05). Both groups were dominated by boys (60.71% in healthy group and 84.44% in ADHD group), and compared with the healthy group, boys occurred more in the ADHD group (*p* < 0.05). The DQ scores of children in the ADHD group were (92.14 ± 1.57), which is in the normal range. In addition, there were no significant differences in the age, gender, and DQ scores among the different types of ADHD.

**Table 1:** Demographic and clinical characteristics of the study sample.

Characteristics	Healthy group (*n* = 28)	ADHD group (*n* = 45)	Statistical values	*p* value
Age (years), M (P25, P75)	[4 (4, 5)]	[4 (4, 5)]	Z = −0.614	0.539
Sex, *n* (%)				
Female	39.29%	15.56%	*χ*^2^ = 5.232	0.022
Male	60.71%	84.44%		
DQ^a^, mean ± SD		92.14 ± 1.57		

^a^ DQ is based on children's neuropsychological development and is divided into five dimensions: gross motor, fine motor, adaptability, language, and social behavior. DQ < 70 indicates mental retardation.

### 3.2 ERP results

**Figure 3:**
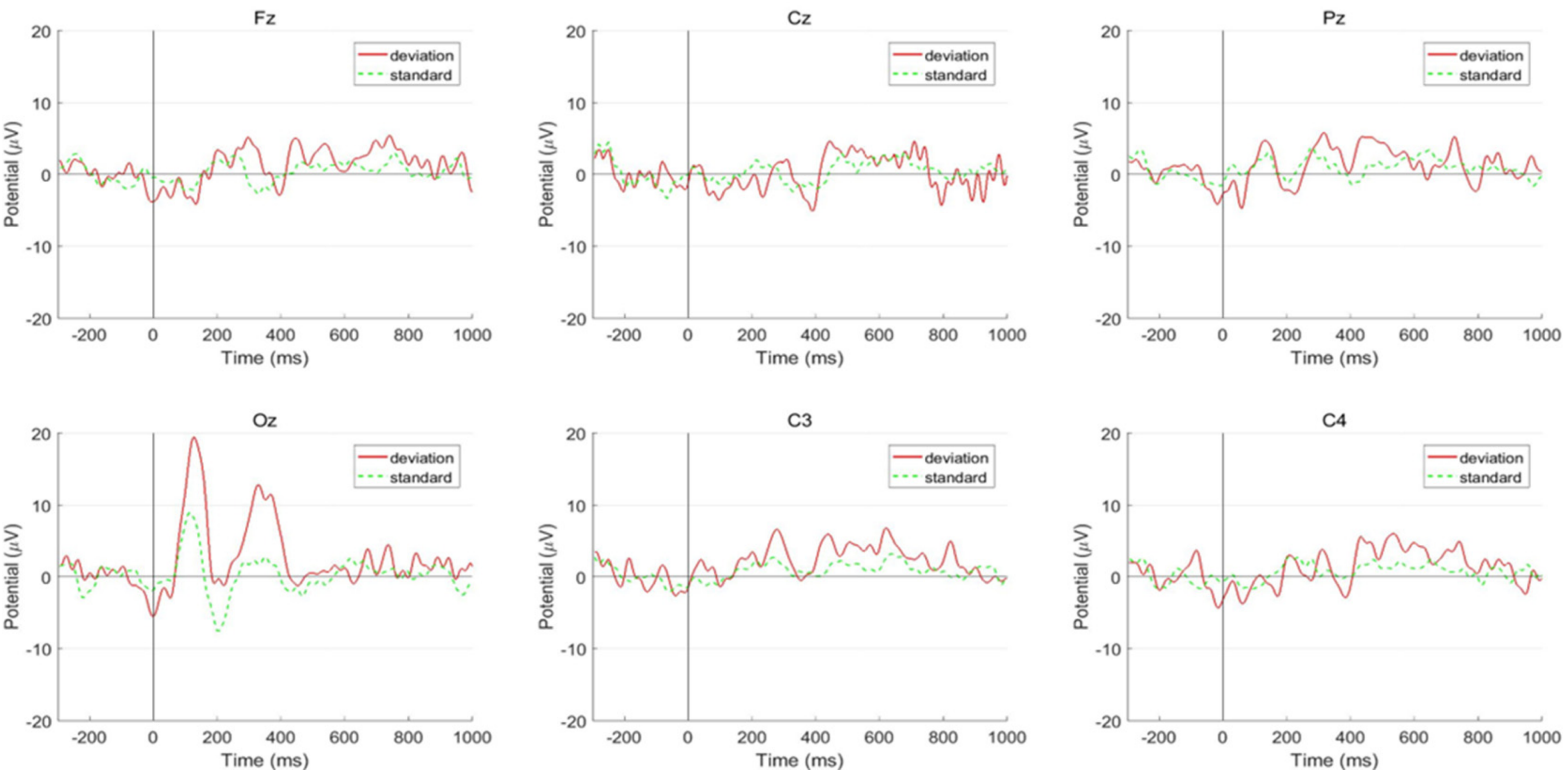
P300 results in controls.

**Figure 4:**
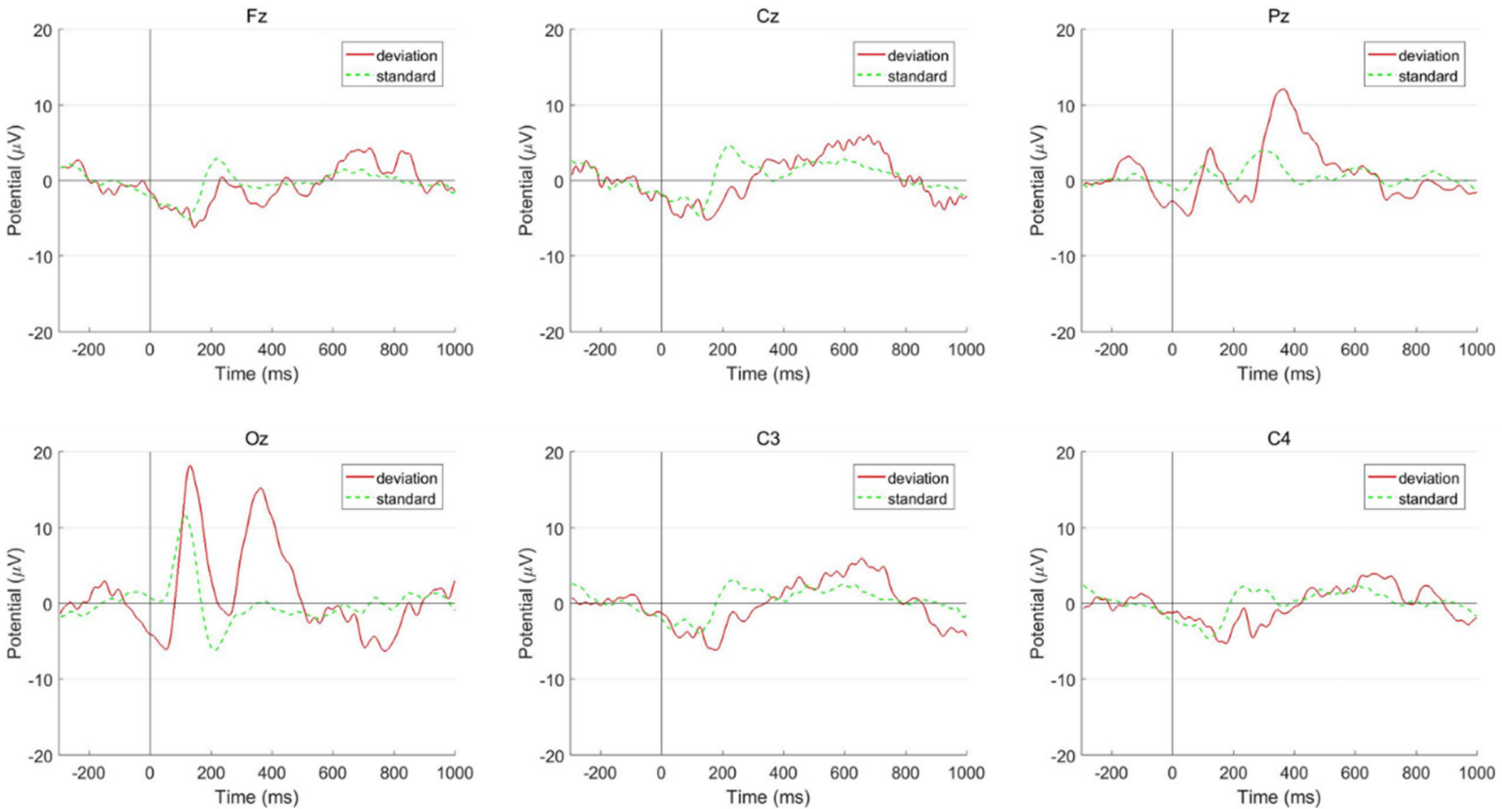
P300 results in ADHD.

The latency of P300 at all the recording electrodes (Fz, Cz, Pz, Oz, C3, C4) in the ADHD group was significantly longer than it in the healthy children group (*p* < 0.05) (Table 2). The wave amplitudes of children with ADHD were significantly higher than in healthy children at Pz and Oz points, meanwhile, the amplitudes at other points (Fz, Cz, C3, C4) were not significantly different between the two groups (Figures 3, 4). In addition, there was no statistically significant difference in ERP results between the three types of ADHD in either latency or amplitude (Table 3), and for children with ADHD, their ERP outcomes did not differ by age or gender.

**Table 2:** P300 results of the study sample.

P300		Healthy group (*n* = 28)	ADHD group (*n* = 45)	Statistical values	*p* value
Fz	Latency	297.28	347.18	Z = −2.666	0.008
	Amplitudes	3.34	5.97	t = −1.569	0.121
Cz	Latency	291.91	367.50	t = −5.060	<0.001
	Amplitudes	4.37	7.19	t’ = −1.833	0.071
Pz	Latency	299.07	359.55	t’ = −5.037	<0.001
	Amplitudes	5.85	12.12	Z = −4.510	<0.001
Oz	Latency	309.34	359.66	t = −5.293	<0.001
	Amplitudes	5.39	12.81	Z = −4.368	<0.001
C3	Latency	290.31	359.02	Z = −3.835	<0.001
	Amplitudes	4.28	5.62	Z = −0.427	0.669
C4	Latency	280.67	373.86	Z = −4.992	<0.001
	Amplitudes	4.79	5.87	Z = −0.476	0.634

**Table 3:** P300 results in different types of ADHD.

P300		ADHD-I (*n* = 2)	ADHD-HI (*n* = 20)	ADHD-C (*n* = 23)	Statistical values (H)	*p* value
Fz	Latency	320.00	307.50	357.69	1.947	0.378
	Amplitudes	4.17	7.20	4.37	2.410	0.300
Cz	Latency	314.00	363.40	373.83	3.900	0.142
	Amplitudes	2.57	7.46	6.04	3.324	0.190
Pz	Latency	333.00	357.20	360.61	0.875	0.646
	Amplitudes	9.56	12.81	11.51	1.213	0.545
Oz	Latency	346.00	348.50	360.13	0.482	0.786
	Amplitudes	8.36	13.78	12.49	1.166	0.558
C3	Latency	319.00	352.15	361.00	2.169	0.338
	Amplitudes	5.12	5.73	3.14	0.333	0.847
C4	Latency	345.00	372.45	401.00	0.581	0.748
	Amplitudes	2.88	5.75	4.28	0.822	0.663

Since there was a significant difference in the ratio of boys to girls between the ADHD and healthy groups, we analyzed P300 results in the boys and girls separately (Tables 4, 5). In boys, there were significant differences in latency between two groups at Fz, Cz, Pz, Oz, C4 electrodes, and significant differences in amplitudes at both Pz and Oz electrodes (*p* < 0.05). For girls’ group, the latency at Cz, C3, C4 are significantly different between ADHD children and healthy children, and the amplitudes at Oz point differ between two groups (*p* < 0.05).

**Table 4:** P300 results in boys group.

P300		Healthy group (*n* = 17)	ADHD group (*n* = 39)	Statistical values	*p* value
Fz	Latency	281.88	335.50	Z = 1.609	0.011
	Amplitudes	3.91	5.84	t = −1.091	0.280
Cz	Latency	305.51	365.15	t = −3.515	0.001
	Amplitudes	4.23	7.05	t’ = −1.842	0.071
Pz	Latency	312.64	361.08	t = −3.406	0.001
	Amplitudes	6.60	12.23	t’ = −4.098	<0.001
Oz	Latency	304.48	358.49	t = −4.964	<0.001
	Amplitudes	6.75	12.21	Z = 1.690	0.007
C3	Latency	282.99	341.50	Z = 1.358	0.050
	Amplitudes	4.61	5.55	t’ = −0.758	0.452
C4	Latency	271.85	378.00	Z = 2.040	<0.001
	Amplitudes	5.02	4.87	Z = 0.945	0.334

**Table 5:** P300 results in girls group.

P300		Healthy group (*n* = 11)	ADHD group (*n* = 6)	Statistical values	*p* value
Fz	Latency	319.69	372.83	t’ = −1.568	0.161
	Amplitudes	2.86	4.17	t = −0.685	0.504
Cz	Latency	288.44	375.50	t = −3.657	0.002
	Amplitudes	3.42	5.71	Z = 0.597	0.868
Pz	Latency	385.19	337.00	t = −1.784	0.095
	Amplitudes	4.95	10.51	t’ = −2.069	0.084
Oz	Latency	316.41	355.17	t = −1.772	0.097
	Amplitudes	4.03	11.30	Z = 1.642	0.009
C3	Latency	300.96	365.50	t = −3.030	0.008
	Amplitudes	4.47	6.08	t = −1.264	0.226
C4	Latency	293.50	384.67	t = −4.452	<0.001
	Amplitudes	4.46	5.49	Z = 0.597	0.868

## 4 Discussion

In recent years, there have been many studies focusing on cognitive function in children with ADHD, however, the application of ERPs in the diagnosis of ADHD is seriously lagging behind. The brain dysfunction of children with ADHD often precedes the diagnosis, so it is essential to identify the clinical symptoms, cognitive functions, and related brain abnormalities in early childhood, which might be the most predictive period for later development of ADHD. Long latency potentials such as the P300 are of use in the study of cognitive and attentional functions, and abnormalities in latency or wave amplitude can indicate the alteration in cognitive processing function of the subject. To the best of our knowledge, the current research is the first to use event-related potential P300 in preschool children with ADHD. Using a cross-sectional study, our study compared and analyzed the characteristics of ERPs in preschool children with ADHD and healthy children to explore the value of P300 for assessment of ADHD in preschool children.

Our study found that preschool children with ADHD had longer latency for P300 at all the electrode sites than healthy controls, which was consistent with the results of other studies in school-age children (23–25). The latency of P300 is considered to reflect the time for the subjects to complete stimulus categorization, with good stability and consistency (26). Generators of the P300 is associated with attentional function-related structures, including the reticular formation, prefrontal cortex, temporal cortex, parietal cortex, limbic system, part of the thalamus and the projection systems between frontal lobes (25, 27). The prolonged latency in ADHD group can be explained by the slower processing of attentional cognitive functions in ADHD children than in normal children. This result indicates that preschoolers with ADHD take longer to process the same stimuli during active attention, which is clinically characterized by attention deficits and extended time required for voluntary attention. Meanwhile, we also found that children in the ADHD group had higher wave amplitudes in the central parietal lobe and central occipital lobe than healthy preschoolers, which contrasts with the findings in school-age children (28, 29). It is generally considered that the amplitude of ERPs reflects the intensity of cognitive resource utilization by the brain: the more resources invested, the higher amplitudes evoked, and there is a positive correlation between the two magnitudes (30, 31). In this study, the reason for the increased amplitude of waves in ADHD children may be that during voluntary-attention progress, more attentional resources were required to be allocated to accomplish the task. Considering the differences in the gender distribution between two groups, an attempt was made to correct for possible bias by statistical methods: we described and compared boys and girls in both groups separately. Almost the same results were found in boys, that is, children with ADHD showed the prolonged latency generally and the higher amplitude in the parietal and occipital lobes. Only the result of latency at C3 differed. Further studies with wider distribution of electrode sites and a larger sample size are required to confirm whether this discrepancy was caused by the lateralization effect. In the case of the girls’ group, the number of subjects was not sufficient enough, but the results still did not appear conflict apparently. We believe that, in future studies, balancing the baseline characteristics properly would make the finding more convincing. Many related studies have obtained prolonged latencies of P300 in ADHD patients, which can be well interpreted as more time required for the brain to process same tasks for children with ADHD. However, studies in the amplitude have yielded different results, including the present research. This may be related to the development of children's own cognitive ability, in addition, the difficulty of each paradigm is different and the “ceiling effect” cannot be ignored. Furthermore, anxiety, obsessive-compulsive, and some other emotional or psychiatric disorders should also be considered. Consequently, how to set up a suitable paradigm for different subjects, or even making individualized programs with adaptive difficulty individually, may be one of the most important directions for future researches. Setting up a queue of regular visits for the appropriate groups is also likely to offer more information.

The results of this study could also provide some information for the clinic: these differences found in younger ADHD patients may suggest that earlier assessment and intervention, such as early cognitive training or medication, may be able to improve the child's condition and prognosis. In conclusion, the results of this study suggest that cognitive impairment has already existed in preschool children with ADHD, which would persist throughout the school age. And the changes in P300 component may provide objective indicators for clinical assessment in preschool children with ADHD.

## Data Availability

The raw data supporting the conclusions of this article will be made available by the authors, without undue reservation.
